# A Complicated Case of Dyspepsia with Hysterical Symptoms, Dependent upon Displacement of the Uterus, Etc.

**Published:** 1853-10

**Authors:** John C. Darby

**Affiliations:** Lexington, Ky.


### WESTERN JOURNAL

OF

### MEDICINE AND SURGERY.

OCTOBER, 1853.

ARTICLE I.—*A Complicated Case of Dyspepsia with Hysterical Symptoms, Dependent upon Displacement of the Uterus, etc.* By JOHN C. DARBY, M.D., of Lexington, Ky.

I was called to see Mrs. K., of ——, on the 21st June, 1853. Mrs. K, has had two children; was in the 28th year of her age. Her last child was born about the 12th of April. From what I can learn, the labor was tedious, and she was bled freely. Why she was bled I do not know. She recovered very slowly from her confinement. Mrs. K. is a tall woman, with a large frame and well proportioned. The pelvic region is remarkably well developed. I found her very much emaciated. The walls of the abdomen had fallen down upon the spine, and made a great depression between the points of the hip bones. The abdominal aorta could easily be felt; the skin covering the abdomen had a tanned or dead appearance ; the bowels were costive at the time, and the secretion of urine was very scanty ; hardly any was passed, and that at intervals of 24 hours ; there was considerable heat over the abdomen, though no soreness on pressure ; the pulse varied from 120 to 140 in a minute, and was weak ; the tongue was remarkably red, large and flat ; nothing agreed with the stomach ; every article of food, and even water, gave rise to pain in that organ. The depression produced after receiving any thing into the stomach was sometimes excessive ; it was always very distressing. The lowness of the spirits of this lady, and her general dejection were very great. Considering her great emaciation, her utter inability to retain any nourishment, her dread of immediate death, and her unmistakable extreme and continual suffering, no one can realise to himself her condition, who had not seen her and watched with her for several days. But for a long practice in chronic disease I would very soon have given up the case as hopeless.

Mrs. K. had been under medical treatment from the time of her confinement. She had been under the care of some four or five physicians, several of whom were among the most distinguished in the State. What was the diagnosis of each, and what the treatment, I do not know with sufficient accuracy to state them. No one had proposed to examine the uterus. She told me that she thought that she was injured at the time of her confinement. She did not reside in Lexington.

At my first visit I made an examination per vaginam. I found the uterus anteverted ; it was not much enlarged; the neck was somewhat elongated and hard. But what struck me as being remarkable was, that the walls of the vagina had fallen back upon the bones of the pelvis, just as if she had not been delivered twenty minutes.

In the first instance I gave her a mild cathartic which was repeated in six or eight hours, as the first dose did not operate. When the medicines operated, the operations were too free. I gave no more cathartic medicine during the treatment, which was up to the 9th of August, except a table spoonful of castor oil at several different times.

I came to the conclusion, after my first examination, that all of the symptoms — of dyspepsia and of hysterics — dependended upon the condition of the uterus and its appendages. I never changed this opinion, and though the distress of the stomach was excessive, I did not believe that it was the seat of her disease ; but that its suffering was symptomatic, or one of the anomalous products of that protean malady—urino-genital disturbance. I tried several remedies to relieve this distress of the stomach, viz.: pills composed of nitrate of silver, gr. $\frac{1}{4}$, opium gr. $\frac{1}{4}$, (opium by itself,) peach-tree leaf water, etc. Neither afforded any relief; inded, they all aggravated her malady. The only things which did agree with her stomach were, ice in small pieces, white sulphur water from Mr. McMurtry's very fine sulphur (bored) well in this city, and effervescing powders of the citrate of potash. But each of these became offensive after a time. The ice agreed with her longer than any thing else. I soon ceased to give any internal medicine whatever. It was a work requiring hours of attention, and of conversation, during the night and day, at times when she was so exceedingly depressed and nervous, to keep hope alive in her, and to make her understand that it required time for the uterus and its appendages to become healthy, and that the dyspepsia, etc., would not be relieved until they did.

The special treatment from the first was as follows: injections with a large glass syringe of a pint or more of cold water, containing two grains of the nitrate of silver to the ounce. The syringe was introduced to near the mouth of the womb. This injection was repeated at intervals for four or five days. After that, a cold solution of alum water was substituted. From the beginning, the vagina was filled up with a cloth wet in cold water. It required as much as a common sized towel to fill it. The patient would remove this cloth whenever it became too hot, have it washed out, re-wet, and then place it back. This was all continued until the walls of the vagina were sufficiently contracted for the space within to be pretty well filled by a gum elastic hollow pessary, which was inflated after it was introduced.

This pessary was about the size of a large hen egg. It was first used about the 10th of July. I omitted to state that the alum water injection was used for some time twice a day; after that, only once a day. The pessary was not worn all the time, it sometimes causing inconvenience, when it was omitted for a while. I recommended my patient to continue the alum water injections, and to wear the pessary, until she again became pregnant. Before she left Lexington the vagina had become so contracted that she found great difficulty in introducing the pessary with the air pressed out. The uterus had assumed its natural position sometime before this.

After the bowels became quiet from the effects of the two doses of cathartic medicine, given when first called in, I found that they again became costive. There was no operation from the bowels for several days. The condition of the stomach was so distressing that I was unwilling to give any medicine whatever, especially cathartic medicine. I then examined the rectum, I found it, as far as it was possible to be, in the same condition in which I had found the vagina; the walls of the bowel had fallen back as far as they could, so as to leave a space in the concavity of the sacrum large enough to hold a hen's egg. The feces were collected in this hollow, and the patient had not the physical strength to cause them to rise up and pass out of the anus. I had to remove them with my finger. This operation I had to repeat frequently.

From the time I made this discovery of the condition of the rectum I had cold water injections thrown into the bowels twice a day for some time, then once a day only until they acted as in health.

The urine began to pass more readily from the time I commenced using the injections, and putting the cold cloths into the vagina. It passed freely long before the bowels began to act healthily. It was at first very high colored. I did not have it analyzed. To correct it I prescribed:

℞.—Phosphate ammonia, ℥ss.

Distilled water, ℥iv. Misce.

Dose, a table spoonful three times a day.

It was not taken regularly, but before it was all taken the urine had become clear and healthy. Whether the medicine effected the change or not I cannot say.

To relieve the distress of the stomach I kept cloths wrung out of cold water constantly applied over the abdomen, with a dry cloth over the wet one. These were renewed as often as they became too hot. They were kept on day and night, and the patient frequently changed them herself, as she said they afforded her more comfort than any thing else. But this distress was so annoying that it seemed as if something more must be done; I therefore applied a blister plaster over the stomach on the 24th of June. It did not draw well and soon healed. On the 29th of June I cupped her over the stomach. Neither the blister nor the cupping relieved the stomach to much extent, and the relief was not permanent. Some days after this I painted the entire abdomen with the tincture of iodine. This was repeated several times, and, I believe did her much service. The painting was speedily followed by a crop of pustules. After a few days the pustules appeared upon her arms and lower limbs. I am inclined to believe that the pustules were produced by the iodine, for she complained of the specific effects of iodine in her throat. The wet cloths were continued all this time, and indeed until she was able to get up.

For some weeks Mrs. K. was unable to sit up at all, and had to be taken up in the arms of a friend and laid on another bed while her own was made up. The improvement in her case was very slow at first, in fact, hardly perceptible. But step by step, as the organs within the pelvis began to contract, and to assume their healthy functions, did the stomach become more quiet, and the hysterical symptoms to pass off. But for some time her digestion was attended with more or less pain or uneasiness. I advised her to eat, however, even at the expense of this distress. The stomach had to become accustomed to its office, as had her limbs when she made the first efforts to walk.

But after a time my patient could eat any thing, tomatoes, beans, boiled corn, meats, etc., and fruits of all kinds, and the difficulty soon was to prevent her from eating too much. As soon as her stomach would bear it, I commenced giving her Scotch ale and London porter. She drank from a half to a whole bottle a day.

I dismissed her cured on the 9th of August. She was then gaining in weight half a pound a day.

So far as I may be entitled to credit for the cure of this lady, I think that I have made it very evident, from a simple statement of the facts, that it all depended upon having made a correct diagnosis when first called to see her. As before remarked, it was extremely difficult to keep hope alive in her, while I waited upon the slow operation of the means applied to the pelvic organs. But all depended upon maintaining the confidence, and not departing from the course adopted.

